# Autophagy in intestinal fibrosis: relevance in inflammatory bowel disease

**DOI:** 10.3389/fphar.2023.1170436

**Published:** 2023-06-15

**Authors:** Dulce C. Macias-Ceja, María D. Barrachina, Dolores Ortiz-Masià

**Affiliations:** ^1^ Departamento de Farmacología and CIBER, Facultad de Medicina y Odontología, Universitat de Valencia, Valencia, Spain; ^2^ Departamento de Medicina, Facultad de Medicina y Odontología, Universitat de Valencia, Valencia, Spain

**Keywords:** fibrosis, autophagy, intestinal fibrosis, inflammatory bowel disease, colitis, Crohn’s disease

## Abstract

Chronic inflammation is often associated with fibrotic disorders in which an excessive deposition of extracellular matrix is a hallmark. Long-term fibrosis starts with tissue hypofunction and finally ends in organ failure. Intestinal fibrosis is not an exception, and it is a frequent complication of inflammatory bowel disease (IBD). Several studies have confirmed the link between deregulated autophagy and fibrosis and the presence of common prognostic markers; indeed, both up- and downregulation of autophagy are presumed to be implicated in the progression of fibrosis. A better knowledge of the role of autophagy in fibrosis may lead to it becoming a potential target of antifibrotic therapy. In this review we explore novel advances in the field that highlight the relevance of autophagy in fibrosis, and give special focus to fibrosis in IBD patients.

## Introduction

Fibrotic diseases are characterized by endless inflammation, tissue wounds, and injuries. Fibrosis is common to various chronic inflammatory diseases in organs (lung, intestine, liver, kidney, or skin) and is responsible for a death rate of close to 50% in developed countries (W.-J. Zhang Wen-Juan et al., 2021). Fibrotic tissues show a decrease in parenchymal cells parallel to an increase in fibrous connective tissue. The fibrotic process is orchestrated by specialized pro-resolving lipid mediators, cytokines, chemokines and growth factors released by immune and non-immune cells. Alterations in metabolic pathways in activated fibroblasts are thought to be responsible for excessive extracellular matrix (ECM) production. The role of autophagy in the fibrotic process is dual and can be bidirectional. To study the profibrotic or antifibrotic role of autophagy, it is necessary to consider several parameters, such as the specific cell type analysed, the stimuli used, the experimental model and the stage of the fibrosis process, as well as to differentiate between the autophagy-mediated effects of secondary compensatory reactions produced by impaired autophagy. In this review we provide insight into recent advances in the field that suggest the implication of autophagy in fibrosis, with special focus given to the fibrosis observed in inflammatory bowel disease (IBD) patients.

IBD is divided into two types: Crohn’s disease (CD) and ulcerative colitis (UC). The incidence of these pathologies in the adult population seems to have stabilized in the western world, but is increasing in paediatric populations and in previously low-incidence areas in developing countries. Both UC and CD share some clinical and demographic characteristics, but differ in the tissue damage they produce and their prognosis. UC is characterized by inflammation and ulcerations that affect the mucosal and submucosal layers of the colon. In contrast, the lesions in CD are common in the distal ileum and right colon, but can affect any part of the gastrointestinal tract. CD is characterized by transmural inflammation (ileal CD accounts for about 80% of cases) and inflammation of the colon (about 20% of cases) (Ng et al., 2017). Intestinal fibrosis is a common and serious complication of IBDs, including CD and UC. Despite the introduction of new biologic therapies, the incidence of stricture formation and stenosis of the intestine in IBD patients has not been reduced significantly (D’Haens et al., 2022; S et al., 2022). As we will detail in the following sections, IBD pathology is mediated by several perturbations related with a defective autophagy, including a malfunctioning mucosal barrier, dysregulation of the immune response, altered microbiota composition and, eventually, the fibrotic process (Foerster et al., 2022), the main focus of this review.

Autophagy is an intracellular pathway that involves the degradation of cytosolic portions, protein aggregates, and organelles through their delivery into lysosomes (Noboru Mizushima et al., 2008). It is vital for the homeostatic turnover of aged proteins and organelles and cell differentiation. In addition, autophagy is activated under situations of low energy and cellular stress, and it is finely regulated by hormones, nutrients and environmental conditions. Therefore, it is not surprising that changes in autophagy modulate the pathogenesis of multiple diseases, including fibrotic diseases. The literature describes three different types of autophagy depending on how substrates are deposited in lysosomes for removal (macroautophagy, microautophagy, and chaperone-mediated autophagy). Macroautophagy (autophagy), the classic, most common type, involves nucleation, membrane modification, development of autophagic lysosomes, and destruction of the enclosed molecules. In mammals, autophagy is initiated by the Unc-51-like kinase-1 (ULK1) complex (Zachari y Ganley, 2017), which¸ together with the class III phosphatidylinositol 3-kinase (PtdIns3K) complex (Kishi-Itakura et al., 2014), contributes to the assembling of the autophagic machinery in the endoplasmic reticulum (ER). Complexes of autophagy-related gene (ATG) proteins, including ATG5, ATG12, and ATG16L1, are attached to the autophagosomes for “maturation” (N. Mizushima et al., 1998). Target molecules can be labelled by ubiquitin tags, as well as cargo receptors, such as CALCOCO2/NDP52 and SQSTM1/P62 (Noboru Mizushima, 2018). After lysosomal fusion has occurred, the contents are digested, and nutrients are recycled (Noboru Mizushima, 2018). The onset of autophagy is closely controlled by various members of the mTOR pathway, such as MTORC1, AMPK and TFEB/TFE3 (Ramirez Reyes, Cuesta, y Pause, 2021). That said, the control of autophagic flux can be regulated in many other ways. In the case of specialized forms of autophagy, including xenophagy and mitophagy, nucleotide binding oligomerization domain containing 1 (NOD1) and NOD2 can begin autophagy through direct interaction with ATG16L1 upon detection of bacterial infection. Then there is the case of miRNAs, which can regulate autophagy through different molecular pathways (NOD2, ATG16L1, ULK1 or ATG9) (Foerster et al., 2022; S. Wang Shiyuan et al., 2018)

Diverse review articles have analyzed the role of autophagy in various fibrotic tissues, such as the lung, liver or kidney, but none have focused on the specific field of IBD fibrosis and how it is related to autophagy process. Existing reviews on autophagy and IBD does so either from the pathophysiological point of view of the disease or are based on acute models of colitis or human samples from patients with non-fibrotic IBD. The analysis of the interaction between autophagy and intestinal fibrosis will help to develop new molecular targets of IBD fibrotic therapy. The present review aims to summarize the evidence of the role of autophagy in the initiation, development, and maintenance of intestinal fibrosis in IBD.

### Material and methods

A comprehensive search of MEDLINE/PubMed, Cochrane Library, CINAHL, Scopus, and Embase was conducted in May 2023. In all cases the keywords “fibrosis and inflammatory bowel disease and autophagy” were employed. In Pubmed, 21 results were found, of which 12 were reviews and 9 were research articles. In Scopus, 33 documents were found: 18 reviews, 11 scientific articles, 2 book chapters, 1 editorial and 1 conference. In Cochrane Library we did not find any articles. In CINAHL, 4 documents were found: 1 review and 3 scientific articles. In Embase, we found 57 articles: 27 reviews, 13 scientific articles, 1 short survey, 1 editorial and 15 conference. Duplications were eliminated and the articles that addressed the objective of this work–i.e., to explore the role of autophagy in intestinal fibrosis in the context of inflammatory bowel disease—were selected and reviewed. Research with acute models of colitis were reviewed but not considered in Tables 2, 3

## A brief outline of autophagy in the pathophysiology of IBD

**FIGURE 1:**
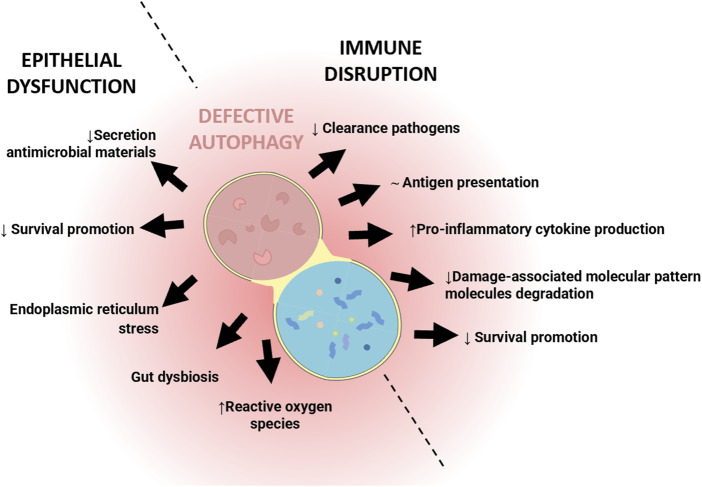
Simplified illustration of the role of defective autophagy in the pathophysiology of Inflammatory Bowel Disease (IBD). The illustration shows the two main processes in which autophagy is involved in the pathogenesis of IBD: epithelial and immune dysfunction.

**FIGURE 2:**
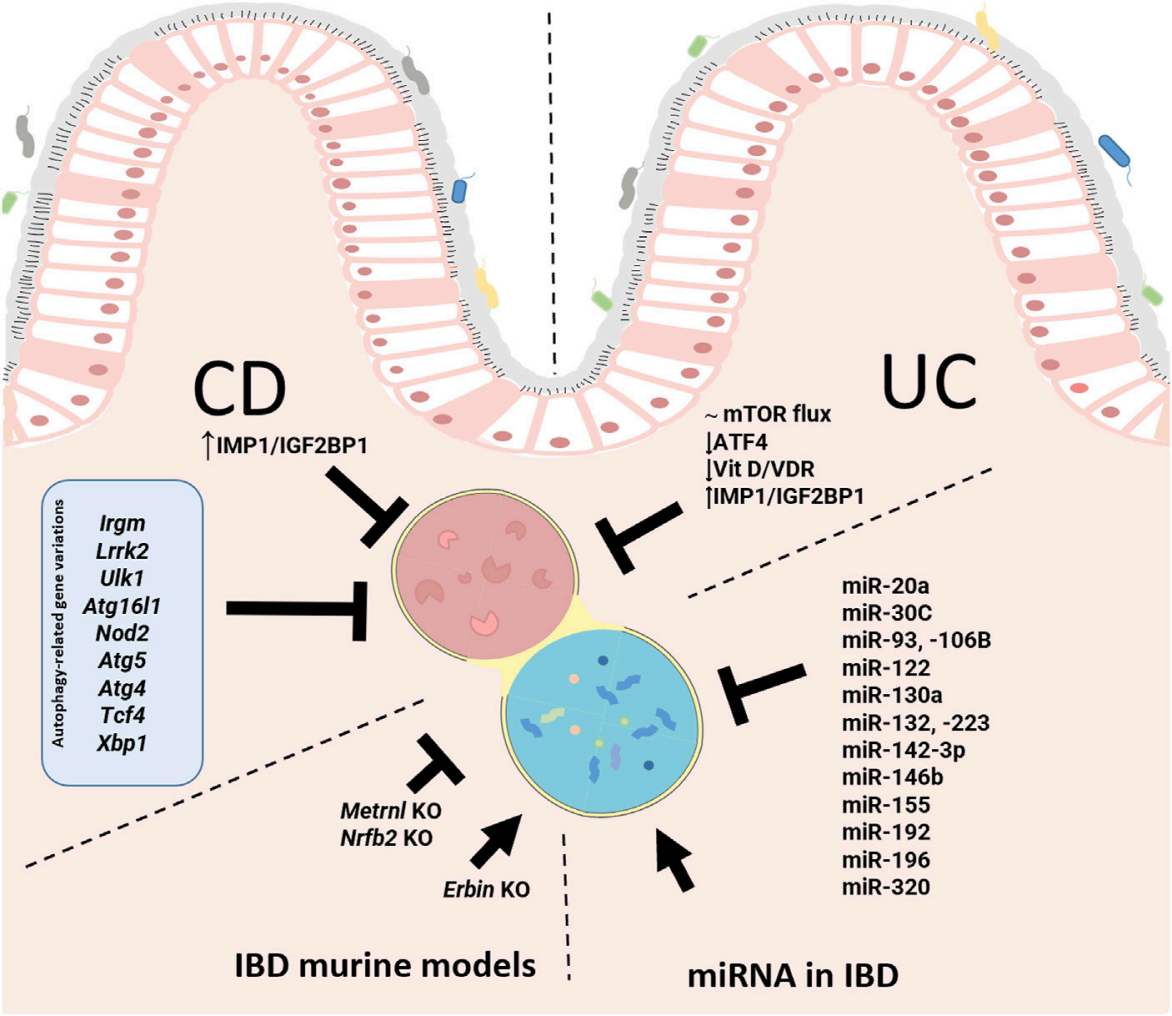
Pathways and genetic and epigenetic basis of autophagy involved in the pathophysiology of inflammatory bowel disease (IBD). In Crohn’s Disease (CD), the mutation or deletion of some autophagy-related genes (blue box) has been related with CD. IGF2BP1/IMP1 has been implicated in autophagy inhibition in CD and Ulcerative Colitis (UC). In UC patients, there is a reduction of the autophagy process through the reduction of ATF4 activity, mTOR-dependent autophagy flux deficiency and low VDR expression and defective vitamin D/VDR signalling. The illustration also features miRNAs described as modulators of autophagy in IBD patients. Finally, we can see the advances made with murine models of IBD that link autophagy to IBD pathology. IGF2BP1/IMP1, Insulin-like growth factor 2 RNA binding protein 1; IRGM, immunity-related GTPase M; LRRK2, leucine-rich repeat serine/threonine-protein kinase 2; ULK-1, Unc-51-like kinase-1; ATG16L1, autophagy-related protein 16-like protein 1; NOD2, nucleotide binding oligomerization domain containing protein 2; ATG5, autophagy-related gen 5; ATG4, autophagy-related gen 4; TCF4, transcription factor 4; XBP1, X box-binding protein 1 spliced-1; mTOR, mammalian target of rapamycin; ATF4, autophagy-related transcription factor 4; VDR, vitamin D receptor; NRBF2, nuclear receptor binding factor 2.

IBD is a multifactorial disease involving factors such as dysbiosis, alteration of the mucosal barrier, genetic mutations and environmental factors. The relationship of autophagy with this disease is widely recognized, both in the early stages and during its development. In general, as shown in Figure 1, alterations in autophagy have been associated with dysfunction of the epithelial barrier, including defects in the secretion of antimicrobial peptides, alterations in the endoplasmic reticulum stress response, dysbiosis, and altered immune responses (Figure 1). Genome-wide association studies (GWASs) have identified IBD-relevant autophagy genes underlying susceptibility to IBD, and especially to CD, which tends to be more affected by genetic factors than UC. Mutation of genes in the autophagy pathway, including immunity-related GTPase M (IRGM) (Parkes et al., 2007; McCarroll et al., 2008), leucine rich repeat serine/threonine-protein kinase 2 (*lrrk2*) (Barrett et al., 2008; Gardet et al., 2010), *Ulk1* (Henckaerts et al., 2011), *Atg16l1* (Hampe et al., 2007), and *Nod2* (Hugot et al., 2001; Ogura et al., 2001), predisposes individuals to severe CD. Cells obtained from CD patients with risk alleles for either *Atg16l1* or *Nod2* cannot initiate the antigen presentation, the autophagocytic process, or bacterial clearance (Hampe et al., 2007; Parkes et al., 2007). In CD patients, the polymorphisms of autophagy-related genes, *Irgm*, *Ulk1* and X box-binding protein 1 spliced-1 (*Xbp1*) imply weaknesses in the initial immune response, principally innate immune pathways and the management of intracellular bacteria (Rioux et al., 2007). Other mutations or deletion of autophagy-related genes linked with CD pathogenesis include *Atg5* in Paneth cells, *Atg4* and transcription factor 4 (*Tcf4*) (S.-L. Wang Shu-Ling et al., 2018). In UC patients, the autophagy process is undermined through reduction of autophagy-related transcription factor 4 (*Atf4*) activity (Hu et al., 2019, 4), mTOR-dependent autophagy flux deficiency (M. Zhou et al., 2018), and a deficient VDR expression and alteration of vitamin D/VDR signalling (Bakke y Sun, 2018) that may modulate intestinal inflammation by promoting autophagy-mediated inflammasome inhibition (Bakke y Sun, 2018; Karimi et al., 2019; Law et al., 2019). IMP1/IGF2BP1, a RNA binding protein which is increased in CD and CU patients, has been implicated in autophagy inhibition. Following dextran sulphate sodium (DSS)-mediated injury, *Imp1* ^*ΔIEC*^ mice show enhanced colonic mucosal recovery, increased autophagy flux and upregulation of ATG5 (Chatterji et al., 2019). In IBD murine models, a protective role of autophagy has been demonstrated through the nuclear receptor binding factor 2 (NFRP2) and Metrnl. NFRP2 regulates autophagy and is necessary for the destruction of apoptotic cells and mitigation of inflammation (Wu et al., 2021). In epithelial cells, deficiency of Metrnl—a secreted protein highly expressed in the intestinal epithelium of UC patients—reduces the autophagy activated by DSS in intestinal epithelial cells and aggravates the disease in DSS-treated mice (S.-L. Zhang Meixia et al., 2020).Over-induction of autophagy can produce negative effects, precipitating autophagic cellular death, for instance. In the DSS model, it has been described that failure of Erbin–a protein implicated in the polarity of epithelial cells, and highly expressed in the intestinal mucosa—produces an over-induction of autophagy and autophagic cell death-triggered inflammation (Shen et al., 2018) (Figure 2).

Autophagy facilitates the maintenance and restoration of gut microbiota homeostasis, and the autophagy-microbiota relationship has also been demonstrated in the pathophysiology of IBD. For instance, xenophagy has been proclaimed to modulate the gut microbiota in IBD. It has been argued that genes related to autophagy, including *Atg7*, *P62, Atg16l1* and *Irgm*, are necessary for the maintenance of intestinal homeostasis through the activation of xenophagy (Shao et al., 2021).

Several studies have implicated epigenetics in IBD pathology, including miRNAs. miRNA is an endogenous non-coding small RNA that performs different biological functions by activating or silencing target genes through complementary pairing bonds involved in the regulation of gene expression. Many miRNAs are known to inhibit autophagy and target IBD-relevant autophagy genes (*Nod2*, *Atg16l1* or *Irgm*), thereby modulating intestinal function and innate intestinal immunity and affecting inflammatory levels in IBD (Jung et al., 2021; S. Wang Shiyuan et al., 2018) (Table 1).

**TABLE 1:** miRNAs related with IBD pathology through modulation of autophagy. ATG16L1, autophagy-related protein 16-like protein 1; SQSTM1, Sequestosome 1; ATG5, autophagy-related gen; PTEN, Phosphatase and tensin homolog; NOD2, nucleotide binding oligomerization domain containing protein 2; NF-κB, Nuclear factor kappa-light-chain-enhancer; m mTOR, mammalian target of rapamycin; FOXO3, Forkhead box O3; SIAH2, E3 ubiquitin-protein ligase; SHIP1, inositol phosphatase; IRGM, immunity-related GTPase M; GSK3B, Glycogen synthase kinase-3 beta; VDR, vitamin D receptor; ORMDL3, Orosomucoid like 3; XBP1, X box-binding protein 1 spliced-1.

miRNAs	Targets	Autophagy	Reference
miR-20a	ATG16L1, SQSTM1	↓	(Liu et al., 2017)
miR-30C	ATG5, ATG16L1	↓	(Nguyen et al., 2014)
miR-93, -106B	ATG16L1, PTEN	↓	N. Li et al., 2017b; C. Lu et al., 2014
miR-122	NOD2, NF-κB	↓	(Yu Chen et al., 2013a, 1)
miR-130a	mTOR	↓	(Yuqiong Wang et al., 2017)
miR-132, -223	FOXO3a	↓	(Kim et al., 2016)
miR-142-3p	ATG16L1, NOD2	↓	(Zhai et al., 2014)
miR-146b	SIAH2, FOXO3	↓	(Nata et al., 2013, 146)
miR-155	SHIP1, FOXO3	↓	X. Huang et al. (2012), Rouquette-Jazdanian et al. (2015)
miR-192	NOD2, NF-κB	↓	(Chuang et al., 2014, 2)
miR-196	IRGM	↓	(Brest et al., 2011)
miR-320	NOD2, NF-κB	↓	(Pierdomenico et al., 2016)
miR-346	GSK3B, VDR	↑	(Yunzi Chen et al., 2014)
miR-665	ORMDL3, XB1	↑	(M. Li et al., 2017a)

## Relevance of autophagy in IBD fibrosis

Intestinal fibrosis is responsible for the high morbidity and surgical rates in patients with IBD. Although inflammation is essential for its initiation, anti-inflammatory treatments do not prevent fibrosis once it has started. Some patients, principally those with CD, develop fibrosis early, and this complication tends to reoccur following resection, whereas this is not observed in other types of patient (Lawrance et al., 2017). Fibrosis in IBD involves the accumulation of ECM components in the intestinal wall by activated cells of mesenchymal origin (fibroblasts, myofibroblasts and smooth muscle cells). ECM deposition is restricted to the superficial layers (mucosal and submucosal) in UC, while fibrosis in CD is located principally in the ileocecal valve and involves the whole bowel wall due to the transmural nature of the inflammation. The main promoters of intestinal fibrosis are soluble molecules (growth factors, and cytokines, of which TGFβ1 is considered a main player) released by activated immune and nonimmune cells, epithelial-to-mesenchymal transition (EMT) or endothelial-to-mesenchymal transition (EndoEMT), G protein-coupled receptors, and the gut microbiota (D’Alessio Silvia et al., 2022; D’Alessio S. et al., 2022). Various autophagy genes, including P62, ATG5, ATG7, ATG16l1 or ULK2, are associated with the regulation of TGFβ1 in lung fibrosis (Ghavami et al., 2018), but little is known about the role of TGFβ1 in the modulation of autophagy in IBD. The role of autophagy in IBD-related intestinal fibrosis is controversial. Many studies suggest it has a protective effect (Holvoet et al., 2017; Cosin-Roger et al., 2019), while others have pointed to the harmful role of excess autophagy in fibrotic processes (Yu et al., 2022). The following section will review the evidence for the role of autophagy in intestinal fibrosis at the genetic, cellular, and molecular levels.

### Genetic and epigenetic basis of autophagy in intestinal fibrosis

Fibrostricture disease recurrence in some CD patients suggests the existence of a predisposing genetic background. Several studies have proposed the relevance of some single-nucleotide polymorphisms related with autophagy. The strongest fibrosis-autophagy association has been established between the variants of the CARD15/NOD2 gene (L1007fsinsC) and ATG16L1 (rs2241880). A fibrostricturing phenotype exhibits a strong association with the activation of autophagy by NOD2 and ATG16L1 in both pediatric and adult CD patients (Fowler et al., 2008; Strisciuglio et al., 2014). In infected cells, NOD2 and/or ATG16L1 mutations can alter the receptiveness of immune cells to bacteria, thus intensifying inflammatory signals that ultimately activate mesenchymal cells to produce ECM (Travassos et al., 2010). Myofibroblasts express NOD2 and ATG16L1 and, through the induction of caspase activation, enhance apoptosis (Luna, Masamunt, Lawrance, et al., 2011a). Apoptosis is responsible for reducing the number of myofibroblasts during fibrosis resolution, so the reduction in apoptosis of myofibroblasts could be extremely profibrotic.

Little is known about the role of miRNA-regulated autophagy in intestinal fibrosis. In neural cells, miRNA-200b activates apoptosis and suppresses autophagy by controlling the expression of the autophagy-related gene Ambra1 (Autophagy/Beclin1 regulator 1) (W. Huang et al., 2021). miRNA-200b has been postulated as a fibrotic biomarker in IBD, since it is overexpressed in the circulation of fibrostenotic CD patients and suppresses *in vitro* fibrosis by inhibiting the transdifferentiation of epithelial cells to myofibroblasts (Yingwei Chen et al., 2012). Alterations in collagen genes give rise to misfolded procollagen proteins which produce toxic aggregates in the ER that can induce ER stress and, consequently, autophagy. This process could be the ultimate cell-protection strategy used to reduce ER-accumulated cytotoxic aggregates in fibrotic tissue. (Del Principe et al., 2013). In IBD, a group of intestinal miRNAs (miRNA-150 (Ciccacci et al., 2016), miR-665 (M. Li Manying et al., 2017), miR-375 (Ambrose et al., 2012) and miR-346 (Guo et al., 2018) appear to modulate autophagy to regulate the unfolded protein response during the ER stress response, thereby promoting intestinal fibrosis in IBD (S. Wang Shu-Ling et al., 2018), though the underlying mechanisms require further validation.

### Autophagy and cellular mediators of IBD fibrosis

**FIGURE 3:**
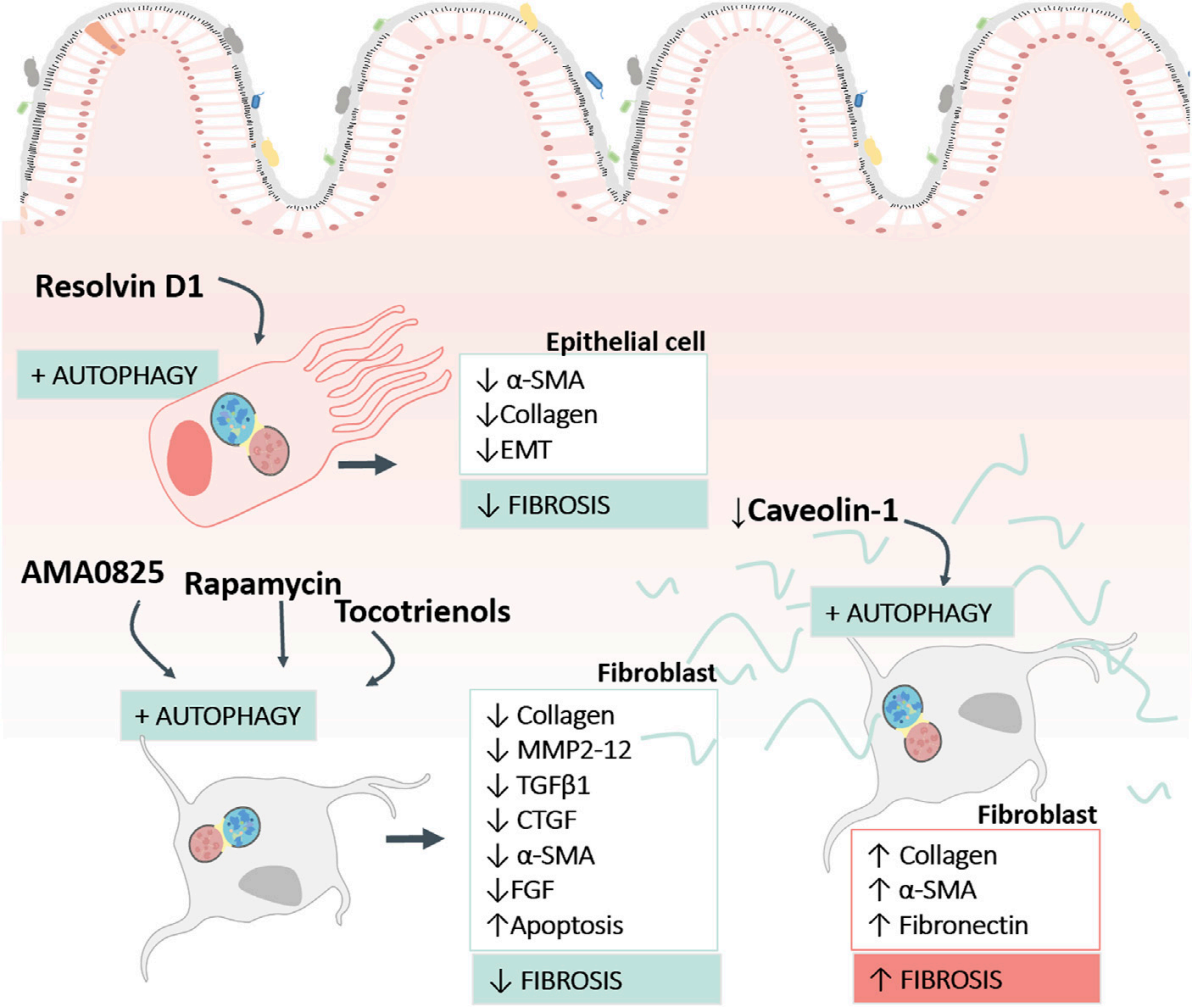
Findings concerning the role of autophagy in human intestinal fibrosis. The image reflects the pro-fibrotic and anti-fibrotic role of autophagy described in human intestinal samples, in function of the cell type (fibroblasts or epithelial cells) and the type of stimulus used to modulate autophagy. The figure also shows the fibrosis markers used to analyse the response. α-SMA, Alpha Smooth Muscle Actin; EMT, Epithelial Mesenchymal transition; MMP, Matrix Metalloproteinases; TGFβ1, Transforming Growth Factor beta 1; CTGF, Connective Tissue Growth Factor; FGF, Fibroblast Growth Factor.

The implication of autophagy in intestinal fibrosis is complicated, as it depends on the cell and the stage of the fibrotic process (fibrogenesis or ECM elimination) in question (Figure 3). Intestinal fibrogenesis is characterized by an accumulation of myofibroblasts (characterized in turn by the expression of α-smooth muscle actin (α-SMA) secretors of ECM proteins and/or an imbalance of proteins responsible for ECM degradation (matrix metalloproteinases (MMPs). Different cell populations, such as epithelial cells; endothelial cells; stellate cells, pericytes; bone marrow stem cells and resident mesenchymal cells (subepithelial myofibroblasts, smooth muscle cells, or fibroblasts) can transdifferentiate into myofibroblasts. The large number of ECM-producing cells in fibrotic tissues appears to be due to increased proliferation, reduced cell death and/or increased cell survival mediated by autophagy.

Currently, the molecular and cellular study of autophagy in the fibrotic tissue of patients with IBD is very limited. Considering that the local mechanisms that maintain the progression of intestinal fibrosis are found in layers deep in the wall, it is preferable to analyse fibrotic intestinal resections before performing a biopsy. Our group (Ortiz-Masiá et al., 2014a) has described impaired autophagy in epithelial cells from the damaged mucosa of IBD patients; we observed a reduction of LC3II, an accumulation of P62 and mTOR proteins, and an increase in P62 staining in epithelial cells in affected tissue obtained from IBD resections. In support of our human data, decreased autophagy has been reported in preclinical models of intestinal fibrosis–the 2,4,6-Trinitrobenzene sulfonic acid (TNBS) model (Zeng et al., 2022) and Dextran sulphate sodium (DSS) model (Butera et al., 2022)—and in a heterologous transplant model (Cosin-Roger et al., 2019) (Table 2).

**TABLE 2:** Summary of the scientific evidence of the anti-fibrotic role of autophagy in IBD. HIF, human intestinal fibroblasts; UC; Ulcerative Colitis; CD, Crohn Disease; IBD, inflammatory bowel disease; TNBS, 2,4,6-Trinitrobenzene-1-sulfonic acid; DSS, Dextran sulfate sodium; TGFβ1, transforming growth factor beta 1; LC3, Microtubule-associated protein 1A/1B-light chain 3; mTOR, mammalian target of rapamycin; ATG, autophagy-related gen; ULK1, Unc-51-like kinase-1; ECM, extracellular matrix; FGF, Fibroblast Growth Factor; PARP, poly-ADP ribose polymerase; COL, collagen; TIMP, tissue inhibitors of metalloproteinases; MMP, metalloproteinases; ITGB6, integrin subunit beta 6; α-SMA, Alpha Smooth Muscle Actin; IL, interleukin; CTGF, Connective Tissue Growth Factor; EMT, epithelial mesenchymal transition; ERK, Ras-dependent extracellular signal-regulated kinase, SNAIL, zinc-finger transcription factor.

	Intestinal fibrotic samples	Treatment	Autophagy		Fibrosis	
(Luna, Masamunt, Rickmann, et al., 2011a)	Primary HIFs from UC, CD and controls	Tocotrienols	(+)	↑LC3II ↑autophagic vacuoles	**↓**	↓ECM proteins (↓procollagen type I and laminin) ↓fibroblast proliferation (↓FGF, ↑apoptosis (CASPASES-3, -8, and -9, PARP))
(Ortiz-Masiá et al., 2014)	IBD samples	-	(−)	↑ P62, p-mTOR ↓LC3II		-
(Cosin-Roger et al., 2019)	Murine heterotopic transplant model	-	(−)	↑ P62 ↓Lc3II/I, Beclin-1	**↑**	↑Col1a1, Col3a1, Vimentin, Tgfβ1, Timp1, Mmp2, Snail1, Snail2, Itgb6
	Murine heterotopic transplant model	3-Methyladenine	(−)	↑ P62 ↓Lc3II/I, Beclin-1	**↑**	↑Col1a1, Col3a1, Vimentin, Tgfβ1, Timp1, Mmp2, Snail1, Snail2, Itgb6
	Murine heterotopic transplant model	Rapamycin	(+)	↓ P62 ↑Lc3II/I, Beclin-1	**↓**	↓Col1a1, Col31, Vimentin, Tgfβ1, Timp1, Mmp2, Snail1, Snail2, Itgb6
	Primary HIFs	TGFβ1		No significant changes (P62)	**↑**	↑Col1A1
	Primary HIFs	Rapamycin/TGFβ1	(+)	↓ P62	**↓**	↓Col1A1
	Primary HIFs	Bafilomycin/TGFβ1	(−)	↑ P62 ↑LC3II/I	**↑**	↑Col1A1
(Holvoet et al., 2017)	Murine DSS model/Myofibroblasts	-		No significant changes (Lc3II/I)	**↑**	↑Il6, Tgfβ1, Mmp2, Mmp3, Mmp8, Mmp9, Mmp12, α-Sma
	Murine DSS model/Myofibroblasts	AMA0825	(+)	↑Lc3II/I	**↓**	↓IL6, TGFβ1, MMP2, MMP8, MMP9, MMP12, α-SMA
	Human biopsies	AMA0825	(+)	-	**↓**	↓MMP2-3-9-12¸TGFβ1, IL6
	Primary HIFs	TGFβ1	(+)	↑ Autophagosomes ↓ P62	**↑**	↑MMP2, IL6, COL1A1, CTGF, TGFβ1 (+) Myofibroblast transition (↓F-ACTIN, VIMENTIN)
	Primary HIFs	AMA0825/TGFβ1	(+)	↑↑ Autophagosomes ↓↓ P62	**↓**	↓ MMP2-12, TGFβ1, CTGF, COL1A1 (−) Myofibroblast transition (↓F-ACTIN, VIMENTIN)
(Butera et al., 2022, 147)	Murine TNBS model	-		↑Lc3II/I	**↑**	↑ α-Sma, TGFβ1, CD147, Ctgf, Col1a2, Col3a1, p-Erk1/2, Il-6, and Il-23, Il-17, Il-13, Il-36, Il-34
	Murine TNBS model	AC-73	(+)	↑Lc3II/I	**↓**	↓ α-Sma, Ctgf, Col1a2, Col3a1, P-Erk1/2, Il-6, and Il-23, Il-17, Il-13, Il-36, Il-34
(Zeng et al., 2022)	Murine DSS model/Intestinal epithelial cells (IEC)	-	(−)	↑ P62, Lc3ii/Lc3i ↓Atg7, Atg9b, Atg14, Ulk1	**↑**	↑ α-Sma, Col1a2, Col3a1 ↑EMT (↓E-cadherin, ↑N-cadherin, Vimentin)
		Resolvin D1	(+)	↓ P62, Lc3II/I ↑Atg7, Atg9b, Atg14, Ulk1	**↓**	↓ α-Sma, Col1a2, Col3a1 ↓EMT (↑E-cadherin, ↓N-cadherin, Vimentin)
	HT-29	Chloroquine	(−)	↑ P62, LC3II/LC3I	**↓**	↑EMT (↓E-CADHERIN, ↑N-CADHERIN, VIMENTIN)
	HT-29	Chloroquine/Resolvin D1	(+)	-		↓EMT (↑E-CADHERIN, ↓N-CADHERIN, VIMENTIN, SNAIL2)
(Arab, Al-Shorbagy, y Saad, 2021)	Murine TNBS model	Dapagliflozin	(+)	↑Beclin1 ↑AMPK/mTOR ↓ P62		↓ colonic apoptosis (↓capase.3, ↓Bax/Bcl2-2) ↓ Inflammation (↓ HMGB1/RAGE/NF-kβ) ↓ROS (↑Nrf2/HO-1)

The intracellular elimination of collagen with the formation of new collagen-based extracellular matrices are necessary for tissue remodelling. In this context, the role of autophagy as a “weapon” to enhance collagen degradation in intestinal fibrosis has been demonstrated in several articles (Table 2). Cosin-Roger and collaborators showed the anti-fibrotic effect of autophagy stimulation in a murine heterotopic transplant model and in human fibroblasts (Cosin-Roger et al., 2019). In a similar way, the local inhibition of Rho kinases (ROCK) (proteins with multiple roles in TGFβ-induced myofibroblast activation) with AMA0825 increases autophagy in fibroblasts, facilitating a reduction in collagen and IL6 production and inhibiting myofibroblast transition (Holvoet et al., 2017) (Table 2). Other molecules with pro-autophagic activity and tested as anti-fibrotic therapeutic options in murine models of IBD include AC-73 and tocotrienols, described as inducers of apoptosis and autophagy. AC-73 is a specific inhibitor of CD147, an immunoglobulin implicated in liver (H.-Y. Li et al., 2015, 147) and intestinal fibrogenesis (Butera et al., 2022, 147) with potential as a marker of IBD in children (H. Wang et al., 2020, 147). On the other hand, tocotrienols, molecules related to vitamin E, have shown great efficiency in the *in vitro* activation of autophagy in intestinal fibroblasts. The treatment was shown to produce an improvement in anti-fibrotic markers in fibroblasts, thus favouring the resolution of fibrosis (Luna, Masamunt, Rickmann, et al., 2011b).

Other drugs in the pre-clinical phase capable of alleviating IBD by modulating autophagy are dapagliflozin and procyanidin A1. Dapagliflozin is a selective sodium-glucose co-transporter 2 inhibitor used for the management of type-2 diabetes mellitus. In the chronic murine TNBS model, dapagliflozin relieves colitis by increasing colonic autophagy and repressing apoptosis through activation of AMPK/mTOR and Nrf2/HO-1 pathways and suppression of the HMGB1/RAGE/NF-κB cascade (Arab, Al-Shorbagy, y Saad, 2021). Procyanidin A1 is a procyanidin (flavonoid) with antioxidant and anti-inflammatory effects. Procyanidin A1 alleviates acute DSS colitis by regulating AMPK/mTOR/p70S6K-mediated autophagy (H. Zhang et al., 2022)

Fibroblast autophagy in fibrotic disorders requires a fine balance, because an excessive autophagy favours cell survival and differentiation/activation (Del Principe et al., 2013). In this line, Yu and collaborators (Yu et al., 2022) have demonstrated the profibrotic role of autophagy as a mediator of the activation of intestinal fibroblasts (Table 3). Using a DSS fibrosis model and *in vitro* studies with primary intestinal fibroblasts, their work shows that Caveolin-1 (CAV1) increases the expression of SQSTM1/p62 and inhibits autophagy, thus preventing fibroblast transdifferentiation and avoiding the accumulation of α-SMA and ECM. Downregulation of CAV1 was observed in fibrotic resections of CD patients, suggesting that CAV1 deficiency induces fibroblast activation through indirect regulation of SQSTM1/p62, thereby promoting fibroblast autophagy. CAV1 is a recognised antifibrotic signalling mediator that inhibits the TGFβ1 signalling pathway in liver (J. Lu et al., 2018) and atrial (M. Zhang Sai-Long et al., 2020) fibrosis.

**TABLE 3:** Summary of the scientific evidence of the role of pro-fibrotic autophagy in IBD.

		Intestinal fibrotic samples	Treatment	Autophagy		Fibrosis	
(Yu et al., 2022)	CD fibrotic human resections		-	(+)	↓P62, caveolin-1	↑	↑ α-SMA, Fibronectin
	Murine DSS model		-	¿?	↓ caveolin-1	↑	↑ α-SMA, Fibronectin, MMP2
	HIFs/CCD-18Co		TGFβ1	(+)	↓ P62, caveolin-1	↑	↑ α-SMA, Fibronectin, CTGF, COL1A1
	Primary HIFs		Overexpression P62	(−)	↑ P62	↓	↓ α-SMA, Fibronectin, COL1A1
	Primary HIFs		siP62/TGFβ1	(+)	↓P62	↑	↑ α-SMA, Fibronectin, COL1A1

In recent years, the contribution of epithelial cells to the fibrotic process has been demonstrated in CD (Lovisa, Genovese, y Danese, 2019; Macias-Ceja et al., 2022; Ortiz-Masià et al., 2020) and UC (Wenxiu et al., 2021). In the TNBS fibrotic model, approximately one-third of (Fibroblast-specific protein 1) FSP1+ fibroblasts are derived from intestinal epithelial cells (Flier et al., 2010). Accumulated data regarding pulmonary fibrosis show that EMT transdifferentiation does not occur completely; rather, the EMT cells act as sources of soluble ligands that favor the transdifferentiation of fibroblasts (Hill et al., 2019; Yue et al., 2022). This phenomenon has also been observed in IBD by Zeng and group, who found that autophagy inhibited the induction of EMT in a HT-29 cell line, and that the co-culture induced fibroblast activation. In the DSS fibrotic model, EMT cells do not move and remain in the original anatomical position (Zeng et al., 2022). Specialised pro-resolving lipid mediators (lipoxin, resolvins, maresins, or protectins) are endogenous lipid autacoids that play a role in the resolution of inflammation, the progression of fibrosis and the regulation of the autophagy mechanism (Recchiuti et al., 2020). In this context, resolvin D1 has been proven to exert an anti-fibrotic effect in liver (J. Li et al., 2021) and intestinal (Zeng et al., 2022) fibrosis by modulating autophagy. In IBD, resolvin D1 reduces autophagy-induced EMT in intestinal epithelial cells and ameliorates intestinal fibrosis by disrupting epithelial–fibroblast crosstalk (Table 2).

### Macrophage autophagy in fibrotic IBD

Various immune cells have been implicated in fibrosis, and among them macrophages play an important role in fibrosis and in the perpetuation of inflammation in the intestine (Ortiz-Masiá et al., 2014; Salvador et al., 2018; Lis-López et al., 2021; Macias-Ceja et al., 2022). Autophagy is also implicated in the innate immune response, as it is involved in the differentiation of monocytes into macrophages, in macrophage polarization, and in several of the immune functions of macrophages (Wen et al., 2022). Macrophages have traditionally been classified as two phenotypes: the pro-inflammatory (or classically activated) M1 macrophage and the anti-inflammatory (or alternatively activated) M2 phenotype, subclassified into M2a, M2b, M2c, and M2d. The macrophage phenotype is constantly changing and adapting in response to new environmental influences. In this context, autophagic control of macrophage polarization could be used as a therapeutic target in fibrosis. Evidence suggests that there is a relationship between the exacerbation of fibrosis, the type of macrophage polarization, and the chronicity of the inflammatory insult. Thus, M1 macrophages initiate the profibrotic process by releasing MMPs and CCL2 that promote EMT/EndoMT and fibrocyte recruitment. Proper functioning of macrophage autophagy is necessary to mitigate chronic inflammation by supressing proinflammatory M1 macrophage polarization (Wen et al., 2022). In the same way, the chronic and persistent activity of M2 macrophages, though tissue repairers, has been shown to favour the overproduction of TGFβ and growth factors that perpetuate the proliferation of myofibroblasts, the activation of EMT/EndoMT and the ECM deposit (Braga, Agudelo, and Camara, 2015).

Different studies suggest that changes in macrophage autophagy modulate the ability of these cells to promote intestinal fibrosis. Macrophages deficient in Atg16L1 show reduced autophagy, which favours the pro-inflammatory role of macrophages (Saitoh et al., 2008). The continued production of pro-inflammatory factors leads to chronic inflammation and, therefore, fibrosis. The murine model of heterotopic transplantation treated with 3-methyladenine (an autophagy inhibitor) favours the development of fibrosis, along with increased expression of proinflammatory mediators and a pro-fibrotic macrophage (CD16^+^), which accumulates in both the fibrotic TNBS model (Salvador et al., 2018) and in fibrotic CD samples (Macias-Ceja et al., 2022). CD16^+^ macrophages express M2 markers and are likely to be the producers of profibrotic cytokines (IL6, IL13, or IL8). Stimulation of autophagy with rapamycin in the murine model of heterotopic transplantation was found to increase the expression of anti-inflammatory mediators, and augmented the influx of macrophages with what seemed to be a regulatory/anti-inflammatory profile (Cosin-Roger et al., 2019). In a similar way, in the TNBS model of intestinal fibrosis, the anti-fibrotic effect of macrophages has been demonstrated, since the stimulation of autophagy favours the inhibition of the IL22/IL23 axis. (Mathur et al., 2019). Besides the traditional mediators classically associated with macrophage function, evidence shows the ability of macrophages to synthesize Wnt ligands in IBD patients. Macrophage-derived Wnt ligands have been shown to induce the EMT process (Macias-Ceja et al., 2022) and to undermine autophagy in the epithelial cells of IBD patients (Ortiz-Masiá et al., 2014).

## Lessons from other organs: Role of autophagy in fibrotic diseases

There is extensive literature analysing the potential role of autophagy in different fibrotic diseases, such as the liver, lung, heart and kidney. All have one element in common, and that is the complex role of autophagy in the regulation and progression of fibrotic disease. Great strides have been made in characterizing the role of autophagy in fibrosis-related processes, such as cell signalling, elimination of misfolded proteins, lipid metabolism, and inflammation progression. When drawing conclusions about the pro-fibrotic or anti-fibrotic role of autophagy, it is necessary to consider the specific cell type analysed, the stimuli used, the experimental model, and the stage of the fibrosis process, and to differentiate autophagy-mediated effects from secondary compensatory actions. In the case of autophagy-mediated effects, we found evidence of how autophagy favours or prevents fibrogenesis by acting directly on cells/processes closely involved in the genesis of fibrosis (myofibroblasts, EMT, EndoEMT or fibroblasts). For example, some studies have shown that autophagy is involved in lipid droplet digestion and energy supply for fibroblast activation and may aggravate fibrosis. However, in the line of secondary compensatory effects we found evidence of cells/processes related to the control of chronic inflammation (cytokines, chemokines, growth factors or specialized pro-resolving lipid mediators released by immune and non-immune cells) which are responsible for orchestrating fibrosis processes. In this sense, some studies have shown that activated autophagy can attenuate inflammatory responses and ameliorate fibrosis in multiple organs. In the following section we will cite the main findings regarding the pro-fibrotic and anti-fibrotic role of autophagy in the development and perpetuation of fibrosis in other tissues, such as pulmonary, renal and hepatic fibrosis, areas in which research is more advanced.

### Anti-fibrotic roles of autophagy in other fibrotic diseases

Among the mechanisms by which autophagy exerts a protective role we can cite lipid accumulation, RNA-binding proteins, anti-inflammatory effects, anti-aging effects, ECM clearance or the Microtubule-associated protein 1S/transcription factor EB (MAP1S/TFEB) pathway. In relation to liver fibrosis, different studies support that the activation of autophagy protects against fibrosis through different pathways, such as the promotion of lipid droplet regeneration, downregulation of pro-inflammatory cytokines in myofibroblasts, or the inhibition of epithelial stellate cell activation thorough upregulation of autophagy-related signalling pathways [Yes-associated protein (Ge et al., 2017), GATA binding protein 6 (Z. Zhang et al., 2017), MAP1S (W. Li et al., 2016) or class III phosphatidylinositol 3-kinase/homotypic fusion and protein sorting complex (Y. Li et al., 2020)]. Downregulation of MAP1S has been described in both hepatic and renal fibrotic tissue, and leads to autophagy-dependent impaired fibronectin clearance and fibronectin accumulation (Y. Li et al., 2020). In addition, the protective role of autophagy at the renal level has been demonstrated in other models, such as rapamycin, Beclin1 ^+/−^, LC3^−/−^(Ding et al., 2014)^,^ ATG5-KO (Kimura et al., 2011) or ATG7-KO (Kawakami et al., 2015), where autophagy helps to control the accumulation of collagen and cytokines, cell infiltration, inhibition of EMT processes and reactive oxygen species clearance (Dai et al., 2022).

Numerous studies have shown that both autophagic function and autophagic flux are inhibited in idiopathic pulmonary fibrosis. Deficient autophagy in idiopathic pulmonary fibrosis inhibits fibroblast apoptosis (Romero et al., 2016; Wei, Sinha, y Levine, 2008) and promotes ECM deposition (Ricci et al., 2013; X. Zhang Xiaohuan et al., 2021), EMT/EndoEMT (Singh et al., 2015; Hill et al., 2019), myofibroblast transformation (Bernard et al., 2014; Im, Hergert, and Nho, 2015), and TGFβ1 signalling pathways (Patel et al., 2012; Ghavami et al., 2018). The protective role of the EB transcription factor, an inducer of autophagosome-lysosome fusion, has been demonstrated in both pulmonary and hepatic fibrosis. Overexpression of TFEB or trehalose treatment controls levels of inflammatory cytokines and prevents fibrosis-attenuating lysosol dysfunction. Currently, numerous drugs are authorized for the treatment of pulmonary fibrosis; although their exact mechanism of action is still not fully understood, it has been found that pirfenidone (Kurita et al., 2017) and nintedanib (Rangarajan et al., 2016) can activate autophagic activity in the fibrotic process.

### Pro-fibrotic roles of autophagy in other fibrotic diseases

The detrimental effect of induced autophagy on fibrotic diseases has been widely demonstrated. The main mechanisms by which autophagy is believed to play a profibrotic role are processes such as lipid droplet digestion, non-coding RNAs, response to non-folded proteins, oxidative stress, or TGFβ/Smad pathway activation. In relation to hepatic fibrosis, there are several studies that have shown a link between autophagy activation and hepatic fibrosis, and they have highlighted the following affects: acceleration of the digestion of LDs as an energy source of epithelial stellate cells (Hernández-Gea et al., 2012); upregulation of the levels of several non-coding RNAs related with fibrosis (miR-96-5p, miR-29b, IncRNA XIST, IncRNA NEATI or IncRNA H19) (Y. Li et al., 2020); elevated formation of NLRP3 in epithelial stellate cells during oxidative stress; or mediation of fibrosis-related signalling pathways (PI3K/Akt/Mtor [(Y. Zhou et al., 2020), β-arrestinI/GSK-3β/SNAIL (Tan et al., 2019) or Platelet-derived growth factor-β/TGFβ/Smads (Yinghong Wang et al., 2019)]. Currently, the pro-fibrotic role of autophagy in the lung has been related with activated alveolar macrophages and the unfolded protein response. Macrophages might endorse the migration and proliferation of fibroblasts by activating autophagy, and bafilomycin A1 favours the unfolded protein response and ECM accumulation. Lastly, a pro-fibrotic role in renal tissue has been demonstrated in pharmacological models [chloroquine, rapamycin and 3-methyladenine) and in models with genetic deletions (ATG7-KO (Livingston et al., 2016), ATG5-KO (Baisantry et al., 2016) or mVps34-KO (J. Chen et al., 2013)]. Signalling pathways capable of altering autophagic markers (WNT/WISP1, Myc or CHOP) or autophagosome-lysosome fusion controllers (LAMP2) have been related to fibrotic processes in the kidney (Dai et al., 2022).

## Conclusion and limitations

Intestinal fibrosis is the primary complication of IBD; however, it is also one of the least understood in terms of pathogenesis, and this affects diagnosis and treatment. Numerous studies clearly support the role of autophagy in IBD pathogenesis, and there is increasing evidence that it also plays a role in IBD progression, especially in fibrotic complications in CD patients. However, the evidence summarized herein highlights the controversial role of autophagy in the regulation and development of intestinal fibrosis, about which it is difficult to draw firm conclusions. The role of autophagy in the pathogenesis of intestinal fibrosis is multifactorial, and its biological effects (autophagy-mediated or secondary/compensatory) differ depending on the cell type, stimuli, and the stage of the fibrotic process or the fibrotic model used. Moreover, at present, intestinal fibrosis models do not meet the main characteristics of IBD-associated fibrosis, and most studies use chronic colitis models in mice, a species that is particularly resistant to fibrosis. Another limitation, related in this case to samples, is that very few studies to date have used human intestinal resections, and this is especially relevant considering that fibrosis develops mainly in the deeper layers in CD. Finally, different ways of monitoring autophagy have been employed in the studies we have reviewed, which constitutes a further hindrance to this field of research. Autophagy flux is considered a changing process, involving different stages. Therefore, changes in autophagy flux, as measured by LC3-II/LC3-I and LC3-II/P62 ratios, represent autophagy activity more adequately than autophagy marker expression, but require unified criteria to draw global conclusions about intestinal fibrosis.

Taken as a whole, emerging evidence confirms and advances our understanding of the role of autophagy in intestinal fibrosis. Most research supports the anti-fibrotic role of autophagy activation in intestinal fibrosis, and, though still a controversial topic, autophagy is increasingly viewed as a promising therapeutic target.

Currently, there are no antifibrotic therapies approved for IBD, but several molecules have shown promising results in preclinical studies, such the Rho kinase inhibitor AMA0825, resolving D1, dapagliflozin, procyanidin A1 and tocotrienols. These molecules may be involved in the antifibrotic effects mediated directly by autophagy (activating fibroblast autophagy and promoting collagen clearance, among others) or by secondary compensatory actions of autophagy (control of chronic inflammation, among others).
